# Study protocol: Novel Methods for Implementing Measurement-Based Care with youth in Low-Resource Environments (NIMBLE)

**DOI:** 10.1186/s43058-023-00526-z

**Published:** 2023-11-28

**Authors:** Ruben G. Martinez, Bryan J. Weiner, Rosemary D. Meza, Shannon Dorsey, Lorella G. Palazzo, Abigail Matson, Carolyn Bain, Kayne D. Mettert, Michael D. Pullmann, Cara C. Lewis

**Affiliations:** 1 The Warren Alpert Medical School of Brown University, Providence, RI USA; 2 Kaiser Permanente Washington Health Research Institute, Seattle, WA USA; 3 Department of Global Health, University of Washington, Seattle, WA USA; 4 Department of Psychology, University of Washington, Seattle, WA USA; 5 Department of Psychiatry and Behavioral Sciences, University of Washington, Seattle, WA USA

**Keywords:** Measurement-based care, Community mental health, Youth mental health, Rapid ethnographic assessment, Tailoring implementation

## Abstract

**Background:**

For youth receiving care in community mental health centers, comorbidities are the rule rather than the exception. Using measurement-based care (MBC), or the routine evaluation of symptoms to inform care decisions, as the foundation of treatment for youth with comorbid problems significantly improves the impact of psychotherapy by focusing care and building engagement and alliance. MBC increases the rate of symptom improvement, detects clients who would otherwise deteriorate, and alerts clinicians to non-responders. Despite its demonstrated utility, MBC is rarely implemented with fidelity; less than 15% of providers report using MBC per recommendations. Previous efforts to support MBC implementation have yielded suboptimal outcomes, in part, due to organizations’ challenges with identifying and prioritizing barriers and selecting and developing strategies to overcome them. New methods are needed for identifying and prioritizing barriers, and matching strategies to barriers to optimize MBC implementation and treatment quality to improve youth mental health outcomes in community settings.

**Methods:**

Pragmatic implementation methods will be piloted in four diverse community mental health centers. Methods include (a) rapid evidence synthesis; (b) rapid ethnography; (c) design kits (e.g., kits with disposable cameras, journals, maps); (d) barrier prioritization, and (e) causal pathway diagramming. These activities will generate actionable barriers; subsequently, we will use facilitated group processes to prioritize barriers and develop causal pathway diagrams to match strategies to barriers to create implementation plans that optimize MBC fidelity (Aim 1). We will track strategy deployment for 6 months, then compare MBC fidelity for another 6 months post-implementation with data from 2 years of historical controls (Aim 2). Finally, we will co-design a toolkit for design kit methods with youth and the practice and scientific communities (Aim 3).

**Discussion:**

Optimizing MBC implementation in community mental health centers could transform youth mental health care by ensuring the most pressing symptoms are targeted early in treatment. The discussion section highlights expected challenges and limits to using the five methods, including recruitment and engagement given the high pressure on community mental health settings.

**Trial registration:**

Clinicaltrials.gov. NCT05644756. Registered on 18 November 2022. This trial was retrospectively registered.

**Supplementary Information:**

The online version contains supplementary material available at 10.1186/s43058-023-00526-z.

Contributions to the literature

- Measurement-based care can improve treatment for youth with comorbid mental health problem, but it is rarely done with fidelity
- Previous efforts to support community mental health centers in using measurement-based care with fidelity have had mixed success; tailoring is needed for measurement-based care to reach its full potential.
- This study pilots new methods for generating locally contextualized implementation plans to optimize measurement-based care through rigorous and practical means that center the lived experience of clinic staff and encourage staff to take ownership of the implementation process

## Background

For youth receiving care in community mental health centers (CMHCs), comorbidities are the rule rather than the exception [1, 2]. Using measurement-based care (MBC) as the foundation of treatment for youth with comorbidities significantly improves the therapeutic impact, as it can help facilitate meaningful communication and define treatment focus [3, 4]. MBC is the systematic, routine evaluation of symptoms to inform care decisions [5]. MBC is an evidence-based framework that offers improvements over usual care [5]. Especially in youth, MBC increases the rate of symptom improvement [1], detects clients who would otherwise deteriorate [6], and alerts clinicians to non-responders [6, 7]. Implementing MBC with fidelity requires three elements: (1) *Collect*, or routine administration of measures for symptoms, outcomes, and processes before therapy sessions; (2) *Share*, or clinician and client score review; and (3) *Act*, collaborative reevaluation of the treatment plan [8]. But MBC is rarely implemented with fidelity outside the confines of controlled randomized trials: less than 15% of providers report using MBC per recommendations [9] and disparities in implementation exist [9, 10].

Previous efforts to support MBC implementation in practice have yielded suboptimal outcomes in large part because, as is common in “implementation as usual,” strategies to support MBC implementation are not matched to important contextual factors; instead, they are selected based on personal preference, organizational routine, ISLAGIATT (“it seemed like a good idea at the time”), and other criteria. Research has shown that tailoring implementation strategies to address high-priority implementation barriers can increase MBC fidelity, at least with respect to one element: routine administration of measures symptoms, outcomes, and processes before therapy sessions [11–13]. Our experience suggests, however, that for tailored implementation to realize its full potential, new methods are needed for identifying and prioritizing implementation barriers and matching strategies to high-priority barriers [14].

The Novel Methods for Implementing Measurement-Based Care in Low-Resource Environments (NIMBLE) study seeks to tailor implementation plans to improve MBC fidelity by empowering clinic staff at all levels to identify, prioritize, and address barriers within their clinics. NIMBLE is driven by three aims that test and refine methods to tailor implementation plans intended to improve MBC fidelity in community mental health clinics: Aim 1—to co-create tailored plans for improving MBC fidelity in community mental health centers; Aim 2—to evaluate the effect of tailored implementation relative to implementation-as-usual on fidelity to MBC practices, by comparing clinician’s fidelity to MBC post-implementation planning compared to historical controls; Aim 3—to co-design toolkits for IMPACT methods piloted in Aim 1 via user-centered design workshops.

## Methods/design

### Context

#### IMPACT Center

The IMPACT Center is a collaboration between the Kaiser Permanente Washington Health Research Institute, the University of Washington, and the University of Michigan. NIMBLE is one of three R34-like IMPACT signature projects. The IMPACT Center, funded by the National Institute of Mental Health ALACRITY mechanism [15], will deliver practical implementation science to solve public health problems. IMPACT will refine practical, relevant, and rigorous implementation strategies, methods, and toolkits to accelerate the ability of under-resourced community settings to implement evidence-based practices with fidelity to improve youth mental health outcomes. Toward this end, the IMPACT Center will overcome three challenges: Challenge I—community settings undertaking EBP implementation face dozens of barriers, and existing methods for prioritizing these do not typically draw on the literature or sufficiently engage practice partners; Challenge II—there is little to no guidance for how to match implementation strategies to prioritized barriers; Challenge III—discrete implementation strategies are rarely optimized and often result in costly and complex activities that tax under-resourced settings. The IMPACT center has two cores: a methods core that brings together interdisciplinary expertise in implementation methods, and an administrative core that evaluates the center and meaningfully incorporates stakeholders in IMPACT methods and research.

#### Washington State CBT+ initiative (CBT+)

The Washington State CBT+ initiative (called CBT+) provides an ideal natural laboratory for IMPACT. CBT+ is an academic-community partnership funded by the state’s Division of Behavioral Health and Recovery [16]. Over nearly 15 years, CBT+ has provided training and some organizational support for delivering EBPs for children and adolescents in public mental health, with strong practice community collaboration and leadership. CBT+’s team-based approach to training includes asynchronous and synchronous web-based training, 6 months of phone consultation, and a yearly 1-day advanced training. Training and phone consultation are led by a University of Washington EBP expert or by an experienced CMHC supervisor who co-leads training and aspects of CBT+ using a train-the-trainer approach [17]. Phone consultations focus on clinical support for applying cognitive behavioral therapy (CBT). CBT+ trains approximately 250 clinicians and supervisors yearly and provides organizational support for EBP delivery through ongoing monthly calls for all previously trained supervisors and a yearly in-person supervisor training.

### Design overview

**Fig. 1:**
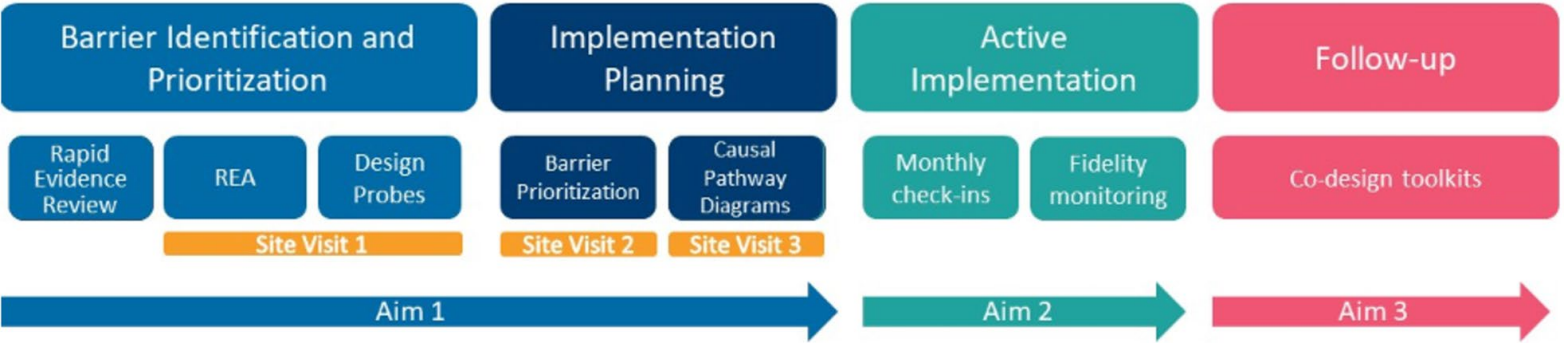
A diagram of study activities. REA, rapid ethnographic assessment

NIMBLE will partner with four clinics connected to CBT+ to optimize MBC use. We will actively seek clinics that are diverse in terms of the following: youth served (e.g., approaching clinics that see primarily Latinx youth), rural/urban status, size, and length of time implementing EBPs. Hereafter, we refer to clinic staff (e.g., clinicians, administrators, supervisors) as practice partners and will specify when referring to a specific group of staff. We will begin by collaboratively identifying and prioritizing barriers, then engage practice partners in developing and enacting tailored implementation plans to address prioritized barriers. We will then track each clinic’s strategy deployment and debrief about the use of IMPACT Center methods to evaluate and refine them. We will check in with clinic implementation teams to support their activities and track strategy deployment once per month; 5–6 months is considered sufficient for active implementation [12, 18]. Finally, we will engage teams in a debrief session to assess the acceptability, feasibility, and appropriateness of IMPACT methods. See Fig. 1 for a visual representation of the study design.

### Aim 1: Co-create plans for optimizing MBC implementation for youth with comorbidities

We will use three complementary barrier identification methods to surface locally contextualized barriers: rapid evidence synthesis (RES), rapid ethnographic assessment (REA), and design kits. Following barrier identification, we will use a group-facilitated barrier prioritization process followed by causal pathway diagramming to tailor implementation plans. Methods will be deployed in partnership with clinics over the course of a year. Ideally, methods will be deployed in-person (Site Visit 1) following a 2-h MBC workshop training for clinicians. However, given that our priority is building and maintaining relationships with practice partners, all study activities could be done in a hybrid or completely virtual format.

#### Barrier identification

Implementation studies usually conduct key informant interviews, focus groups, or surveys to identify barriers [19–22]. Once barriers are uncovered, researchers then typically engage a limited subset of stakeholders in identifying and prioritizing barriers by rating them by feasibility and importance. This approach to barrier identification and prioritization has several limitations: (1) it does not incorporate barriers from the literature; (2) it has issues of recall, bias, and social desirability; (3) it does not sufficiently engage practice partners and youth in evidence-based practices (EBPs) prior to assessment; (4) it does not capture the lived experience of practice partners or youth and typically does not ask about issues related to workflow, decision support, or informatics.

**Fig. 2:**
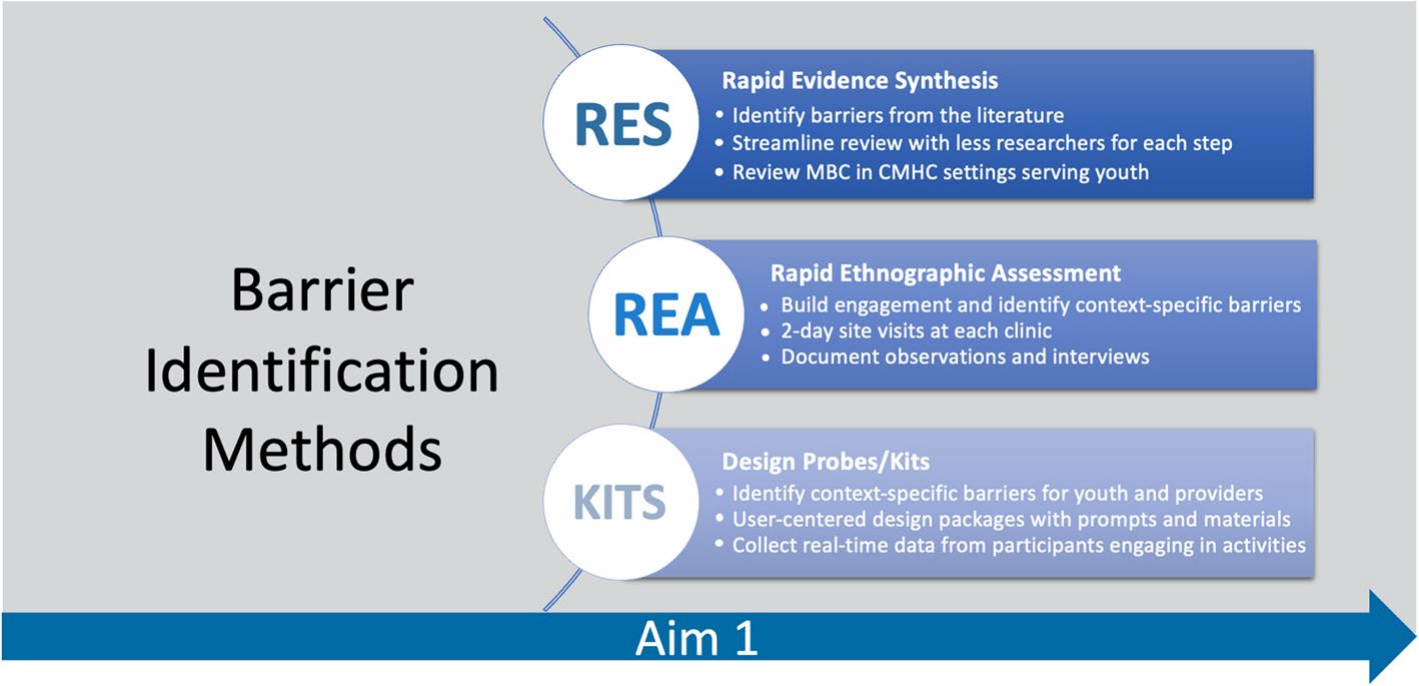
Barrier identification methods

We propose an approach to barrier identification that rapidly and meaningfully centers the voices of those who have historically been least likely to be a part of the implementation process: clinic staff that are not clinicians, supervisors, and administrators. Our approach to barrier identification is multi-method and multi-informant, incorporating scientific literature with promising user-centered design methods (see Fig. 2).

##### Rapid evidence synthesis (RES) to identify barriers from the literature (pre-site visits)

Systematic reviews provide invaluable scientific insight for identifying barriers to implementation. They are, however, very resource-intensive, typically costing around $100,000 to appropriately fund and 2 years to complete [23, 24]. Nimbler methods for evidence synthesis, like RES, promise to ease the burden of these efforts by reducing the resources needed to complete the review process while maintaining scientific rigor [25]. RES can be done in a matter of weeks, rather than months or years, and emerging evidence suggests that the resulting evidence is comparable [26]. Using RES methods may be an important step toward truly being able to incorporate implementation research into practice.

To identify barriers from the literature, we will conduct a rapid review to identify known barriers to implementing MBC in CMHCs serving youth. We will begin with expert recommendations of key articles to focus on pilot searches. We will then identify key terms using PubMed MeSH terms [27] and key terms and synonyms from established reviews (e.g., measurement-based care, implementation, barrier; [5, 28]) to conduct pilot searches of PsycINFO, PubMed, and Web of Science. These pilot searches will document the yield, sensitivity, and specificity of each search and ensure that exemplar articles will be captured within the search. We will systematically search PsycINFO, PubMed, and Web of Knowledge. The systematic search will be supplemented by targeted searches of relevant journals, reference reviews of relevant conceptual papers and systematic reviews, and an informal review of Google Scholar and ResearchGate. Screening will be done in Rayyan, an online systematic review tool [29]. Studies will be included if they (1) surfaced barriers to MBC, (2) were done in mental or behavioral health settings, and (3) focused on youth mental health treatment. The lead author will screen titles and abstracts. Two team members will conduct independent full-text screening and resolve discrepancies by consensus. The lead team member will abstract study data (e.g., setting, population, barriers identified) and distill identified barriers into the levels of analysis (e.g., individual client, organizational) and categories (e.g., attitudes, concerns about breach of confidentiality) identified in the Lewis et al. review [5]. This list of barriers will serve as a conceptual foundation to each subsequent method.

##### Use rapid ethnographic assessment to build engagement and identify context-specific barriers (REA; Site Visit 1)

Barrier identification methods do not typically include a wide range of stakeholders, and when they do, these methods are often in a restrictive survey format or in a brief interview or focus group that is decontextualized. Rapid ethnography uses observational and interviewing methods that seek to understand the experience of those working within clinics “in a rapid timeframe to promote action” [30]. REA is particularly promising for identifying barriers and understanding an implementation context. Developing and refining REA methods for use in community mental health centers was one of NIMBLE’s primary foci.

**Procedure**

REA consists of fieldwork (e.g., clinic site visits) to gather data and build relationships, followed by a synthesis of those data [30]. We will use REA methods over 2-day site visits at each clinic. We will conduct two types of ethnographic observations. First, we will use unobtrusive techniques, documenting observations of activities (e.g., clinician documentation) and significant events (e.g., staff meetings) using written and audio-recorded field notes. Second, we will audio-record semi-structured, focused interviews with as many practice partners as possible. Amongst our team, we will (1) record debrief huddles twice at each visit, where we discuss impressions of the established MBC process, clinic climate, and gaps in our knowledge, and (2) document other relevant observations in writing.

**Participants**

For each clinic, we expect up to 30 clinician and staff interviews: 30–45 min for providers, 15 min for staff. We expect a final sample of *N* = 120 clinic staff to complete interviews.

**Data analysis**

Our team will engage in a rapid analysis approach to document the occurrence or presence of barriers at each clinic, noting duration, time, location, and affected persons, situated in organizational, social, and task contexts that capture the lived experience of practice partners. In the month following each clinic visit, we will triangulate data from (1) unobtrusive observations, (2) notes from formal interviews, and (3) audio and written notes from debrief huddles. We will analyze data using the following steps. First, data will be condensed and reduced into a spreadsheet format. Second, data will be iteratively coded into themes using thematic analysis methods. Coded data will be categorized with the explicit purpose of (1) identifying barriers (what is getting in the way of MBC use), (2) identifying facilitators (are there established strengths we can leverage to promote MBC use), and (3) identifying themes that may aid in developing implementation strategies (strengths and possible strategies). These data will be used to generate a list of clinic-specific barriers to be used in a group-facilitated barrier prioritization exercise with clinic partners. This list will also be shared with practice partners and clinic administration. No direct quotes will be used.

##### Use design kits to identify context-specific barriers (Site Visit 1)

We will develop and use design kits with youth and clinicians to complement REA and collect highly contextualized data that identifies barriers to MBC. Design kits, also called design or cultural probes, are user-centered design packages containing prompts and materials that seek to generate real-time data by asking participants to engage in specific activities [31, 32]. For instance, a design kit activity may ask a clinician to journal about a specific time when discussing measures with a youth client went well. To our knowledge, design kits have never been used in the youth mental health field and have rarely been used in implementation science. Not incorporating user-centered methods and factors in treatment development is a fundamental missed opportunity in psychosocial treatment development, and a problem that implementation science is poised to solve. Because design kits are completed in situ, they promise to unearth important insights about the way practice partners and youth live that are difficult to glean using traditional research methods.

**Procedure**

Clinicians and youth will have 7 days (1 day per activity) to engage in design kit tasks before returning the design kits in self-addressed, stamped envelopes. Within a week of receiving the data, team members will conduct follow-up phone interviews, inviting participants to interpret and comment on their responses.

**Participants**

We will approach all practice partners that do therapy with youth (clinicians, clinical leads, case managers). We expect between 24 and 32 clinic staff across clinics to complete design kits. We will recruit at least 6–8 youth clients per clinic to also complete design kits. We expect between 24 and 32 youths to complete design kits.

**Materials**

**Fig. 3:**
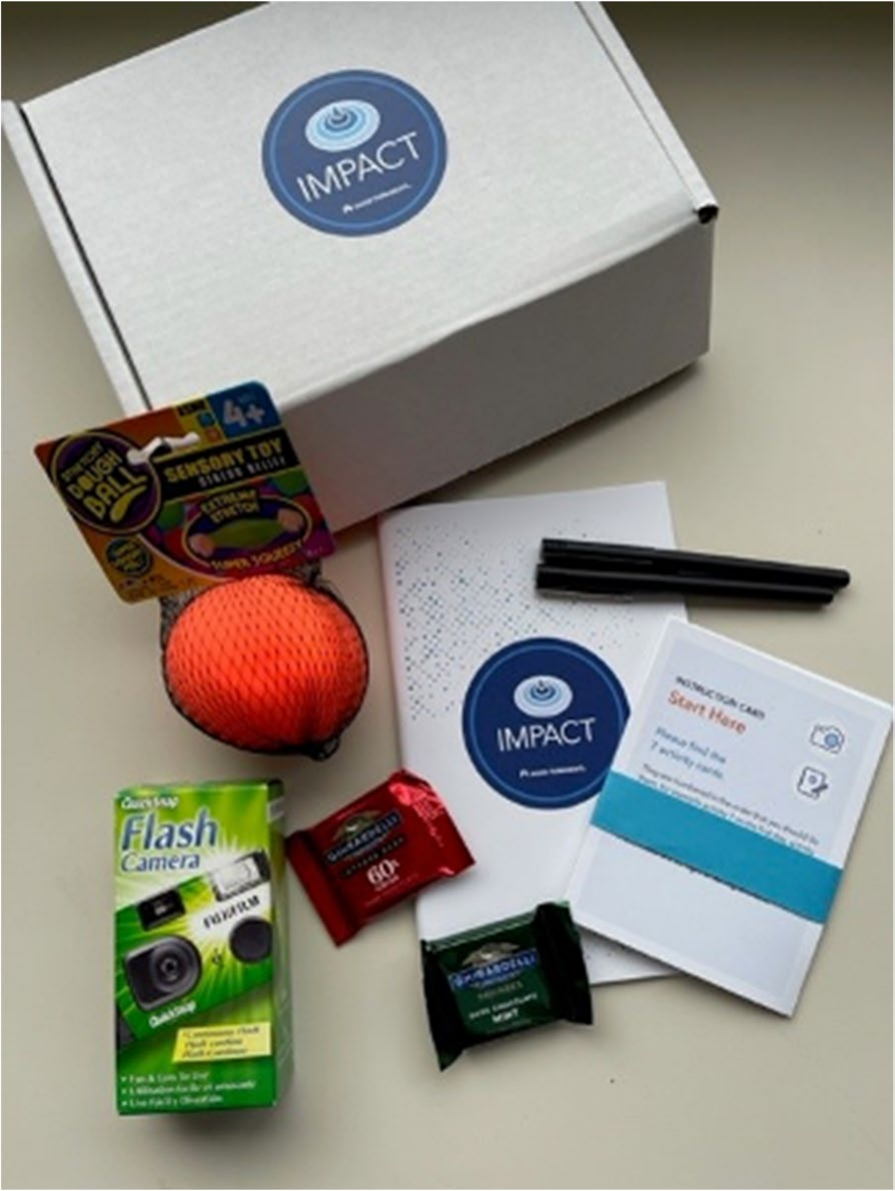
Design kit materials. Materials include seven activity cards and an instruction card, a study information sheet, a journal, two pens, a disposable camera, a stress ball, and two pieces of chocolate

Design kit materials for clinic staff and youth are the same, but the tasks and prompts differ. Each design kit includes a pre-paid return envelope, activity prompt cards, disposable camera, journal, two pens, a small gift (two pieces of chocolate), and stress ball (see Fig. 3). For clinic staff, we focus on prompts that ask clinicians to discuss the role of MBC in their day-to-day workflow. For youth, we intend prompts to be engaging, with some focused specifically on MBC and others focused on the youth’s broader experience in treatment. Design kit prompts are in Supplementary file 1. Design kits were developed in collaboration with the Enhanced Art, Graphics, Literacy, & Editorial Strategies (EAGLES) team at KPWHRI.

**Analysis**

Team members will transcribe the written responses into a text document. Text documents and photos will be used as a basis for creating memos documenting barriers and describing their salience, meaning, and importance to the participant. These barriers will also be incorporated into the clinic report and Barrier Prioritization exercise.

#### Implementation planning

Barrier prioritization that focuses primarily on feasibility may overlook key barriers most closely tied to implementation success [11]. These approaches tend to prioritize individual provider barriers over acknowledging issues for youth (e.g., preference), supervisors (e.g., leadership EBP priorities), team (e.g., workflow), clinics (e.g., decision-making support), organizations (e.g., informatics), or systems (e.g., reimbursement).

**Fig. 4:**
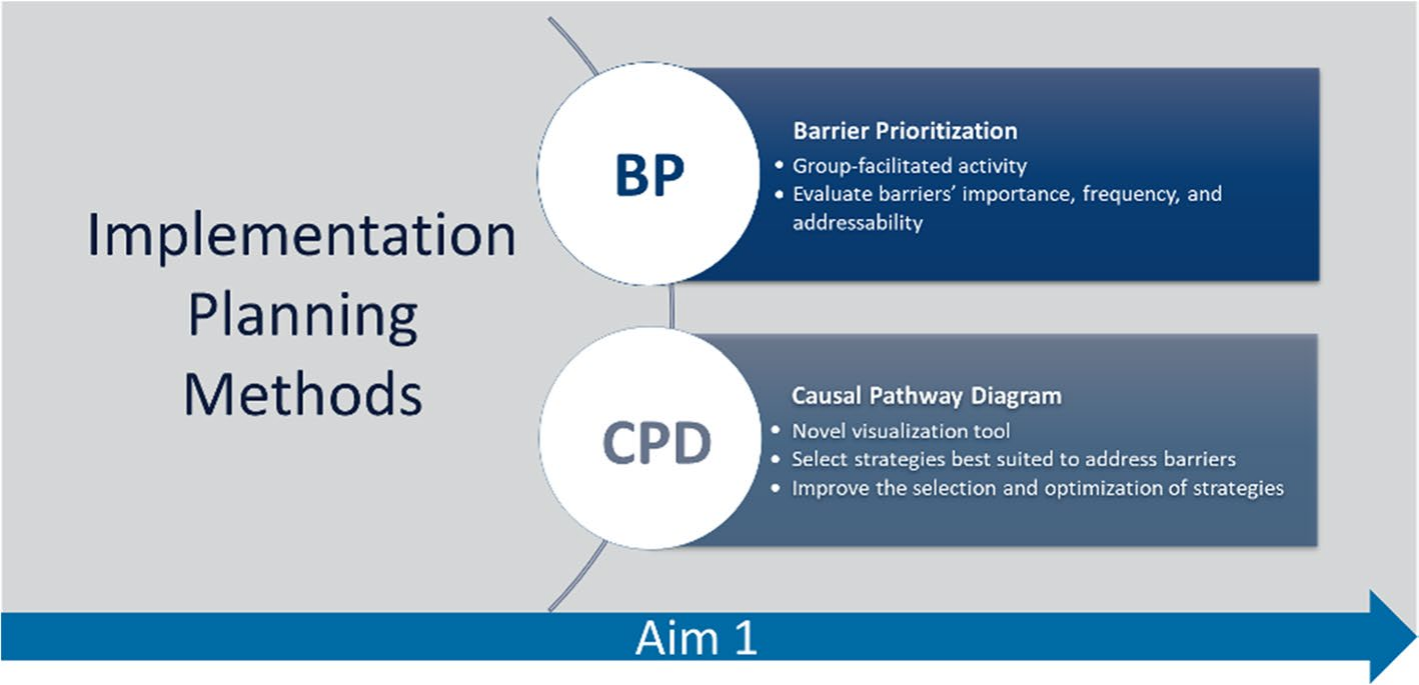
Implementation planning methods

We propose to first recruit an implementation team of 6–8 practice partners per clinic. We will attempt to recruit practice partners that use MBC or have some influence on MBC. To that end, we will approach clinicians and clinical supervisors, as well as clinic leadership and clinic administrators. Our goal is to ensure that the implementation team provides a representative viewpoint and basis for decision-making. The NIMBLE team will pilot two collaborative methods with implementation teams: barrier prioritization and causal pathway diagramming. Figure 4 shows implementation planning methods.

##### Group-facilitated barrier prioritization activity (Site Visit 2)

To prioritize barriers to using MBC with fidelity, IMPACT will pilot a new group-facilitated activity with practice partners. This 2-h group-facilitated activity will occur in-person or virtually using a videoconferencing platform (Site Visit 2).

**Procedure**

We will engage in member-checking [33] by presenting all identified barriers, discussing the findings related to each barrier, and ask participants to reflect on their experience with each barrier. Practice partners will rate each barrier on a 4-point Likert scale on the following criteria: importance, frequency, and feasibility. Importance refers to *how much* the barrier gets in the way of MBC. Frequency refers to *how often* the barrier gets in the way. Feasibility refers to *how feasibly* the barrier can be addressed.

**Participants**

We will engage implementation teams comprised of 5–8 practice partners at each clinic. In total, we expect *n* = 20–32 clinic staff to prioritize barriers.

**Analysis**

We will isolate barriers that are above the mean on all three criteria (importance, frequency, feasibility). To do this, we will (1) calculate the mean rating of each criterion for each barrier, and (2) identify barriers that were scored above the mean on all three criteria. We will present the top five most highly ranked barriers to practice partners, who will collaboratively decide on which three to address.

##### Tailoring implementation plans using causal pathway diagrams (Site Visit 3)

Following barrier prioritization, we will engage with practice partners to create causal pathways diagrams (CPDs) to compare and select implementation strategies best suited to address barriers to MBC use. CPDs are a novel visualization tool to improve the selection, design, and optimization of implementation strategies [34]. They support implementers, including researchers or practitioners, to clarify thinking about how implementation strategies work and under what conditions they work. They rely on theory and experience to capture the implementers’ current understanding of the process through which an implementation strategy is thought to address an identified barrier and bring about improved implementation outcomes.

**Procedure**

**Fig. 5:**
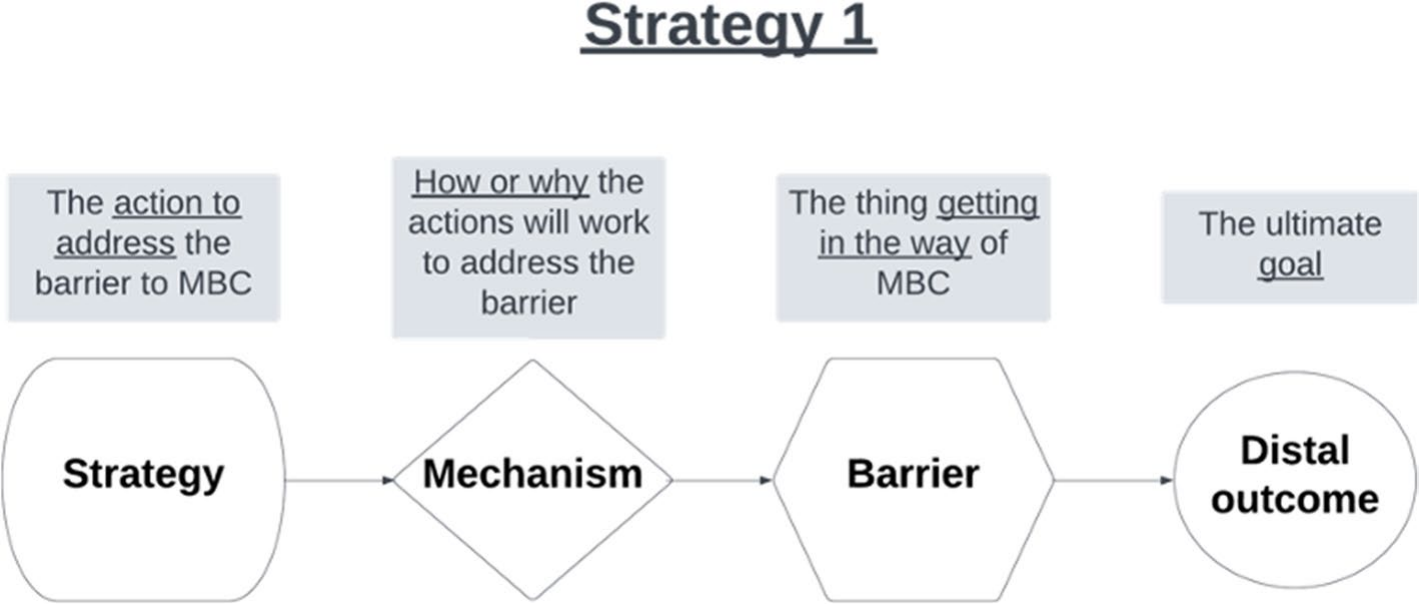
An empty causal pathway diagram

**Fig. 6:**
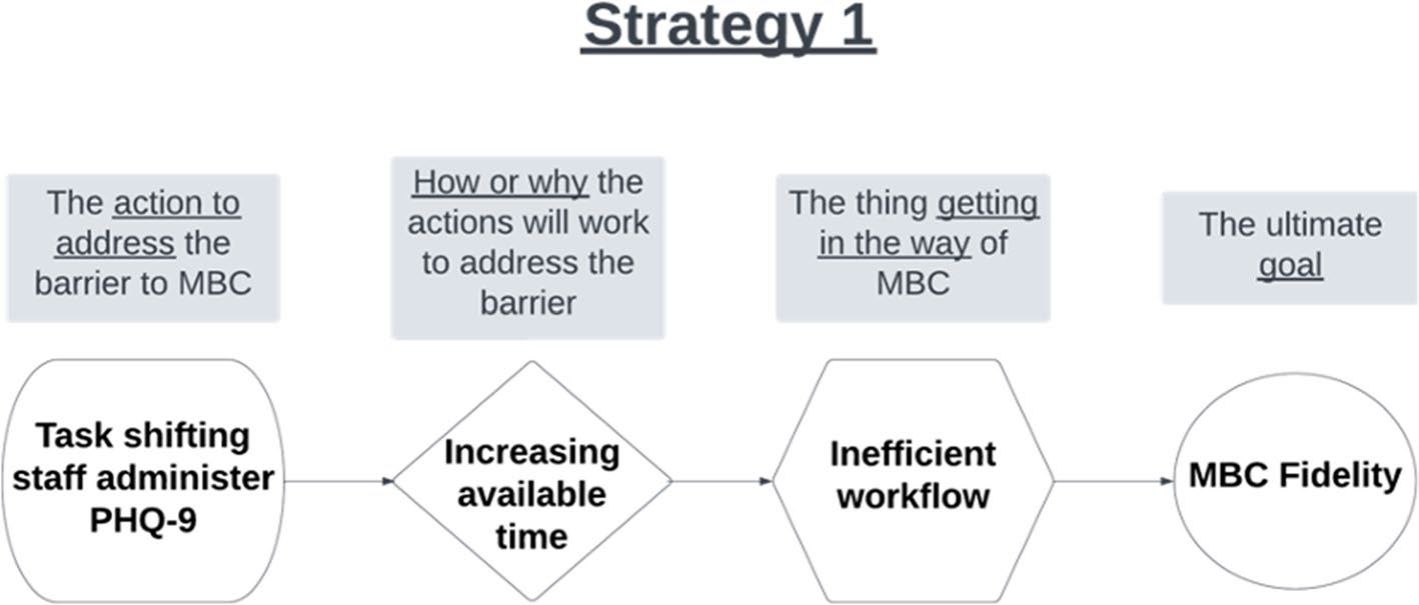
An example completed causal pathway diagram. PHQ-9, Patient Health Questionnaire-9; MBC, measurement-based care

For each clinic, we will develop CPDs for the top 3 prioritized barriers. The goal is to assess how well each implementation strategy is matched to a prioritized barrier, based on its mechanism of action, and to clarify the causal chain of events that must take place to achieve MBC fidelity (i.e., *how* does this strategy work to address this barrier). To illustrate IMPACT’s steps for developing CPDs in Project 1, we have selected an example of the strategy *Task Shifting* to address a possible barrier to MBC fidelity, *Inefficient Workflow*; Fig. 5 depicts an empty CPD, and Fig. 6 depicts an example CPD that articulates the strategy, mechanism, and outcomes for a strategy-barrier pair.

Our research team will develop a CPD for the top barrier in advance of meeting with the implementation team in each clinic who participated in the barrier prioritization activity (Site Visit 3). We will use a diagramming software to build CPDs in real time. We will begin by re-orienting practice partners to the purpose of NIMBLE. Next, we will present the example CPD, introduce practice partners to the construction process, discuss how the CPD aligns with their experiences, and modify it as needed. We will then guide practice partners in collaboratively constructing CPDs for the other barriers using plain-language questions to facilitate the 4-step process described below. This process will result in detailed, co-designed implementation plans.

*Step 1*: The research team and practice partners will collaboratively select at least one promising strategy from existing compilations [35, 36] and our experience, per prioritized barrier. Strategy selection will be guided by an assessment of the evidence, plausibility, and feasibility of each strategy. For instance, strategies that include major overhauls to a system-wide electronic health record are likely infeasible, so a strategy like that would not be selected. We will use Proctor, Powell, and McMillen’s [36] recommendations for strategy specification to help practice partners operationalize each strategy. We will work together to specify the strategies using the recommendations for articulating the actor, action, target, justification, dose, and timing [36].

*Step 2*: We will articulate the mechanism that describes how or why each implementation strategy works. We will use plain language questions (e.g., “How can this strategy change *inefficient workflows?”*) to engage stakeholders in identifying plausible mechanisms. This step aims to ensure that each strategy is aligned with the barrier.

*Step 3*: We will explore the presence of preconditions, or factors that are necessary for a part of the causal chain of events to take place. For instance, if the strategy to address inefficient workflow was to task-shift the administration of outcome measures to the front desk team, a necessary precondition is that this strategy would need to be approved by the necessary administrative person(s) and further require knowledge translation to brief the administering team on the measures and how to discuss them. Preconditions do not always exist, but if they do, we want to account for them to ensure the necessary conditions for an implementation strategy to work are in place.

*Step 4*: Practice partners will rank-order the most convincing, or plausible CPDs. These CPDs will help practice partners select the most promising strategies while serving as detailed plans for their work. After selecting promising strategies and generating CPDs for the three most highly ranked barriers at each clinic, we will discuss with our partners how to deploy these strategies and evaluate their impact, focusing on preconditions, moderators, and outcomes of implementation success. We will follow up virtually monthly via e-mail and/or phone to explore any changes in prioritized barriers, strategy deployment, and MBC fidelity.

**Participants**

We will engage the implementation team of 5–8 practice partners in CPD development. In total, we expect between 20 and 32 practice partners to engage in this activity.

### Aim 2: Compare MBC fidelity post-IMPACT Center method deployment versus historical controls

The goal of tailoring implementation is to improve clinician’s fidelity using MBC. In this case, fidelity will be conceptualized as clinician’s use of the three main components of MBC—(1) Collect, (2) Share, and (3) Act [8]. We will assess changes in fidelity resulting from implementation tailoring activities through (1) clinician self-report and (2) tracking measure use in the CBT+ Dashboard, an anonymous online system for tracking outcome measure use for clinicians that have undergone CBT+ training. We hypothesize improvement in MBC fidelity at 6 months post-implementation.

#### Design

For both methods, we will employ a historical control design comparing pre- and post-IMPACT method deployment from the same clinic, given that contextual factors that directly influence EBP practices, including MBC, vary by clinic. For the clinician self-report, we will ask clinicians to self-report their use of MBC with youth clients before, during, and after the active implementation period. For the CBT+ Dashboard data, we will capture data from 2 years prior to (a) increase sample size and ability to detect significant differences, and (b) account for cohort-specific effects that may influence a particular year. We will ask clinic directors whether any clinical changes may have influenced MBC fidelity during this prior period.

#### Procedures

**Fig. 7:**
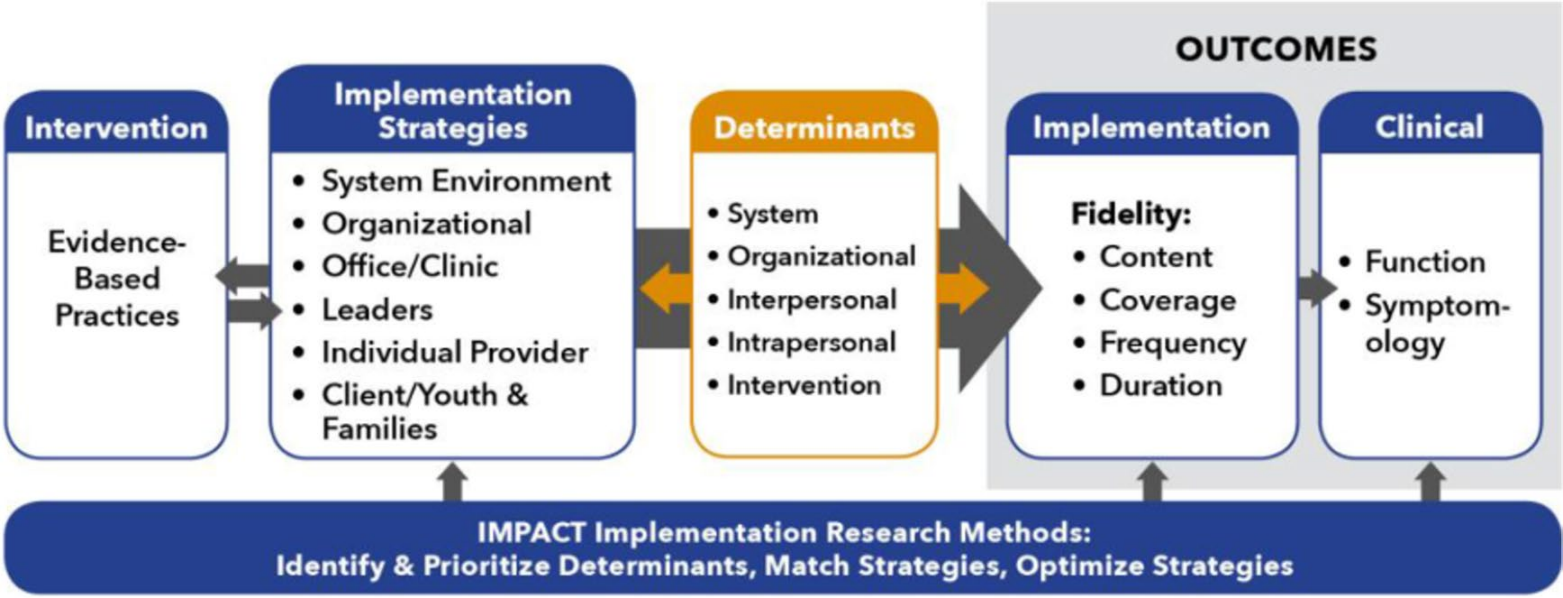
IMPACT quality model (IQM)

We will administer the self-report survey through REDCap [37] before active implementation begins. The active implementation period will begin after implementation plans have been generated and the necessary pre-work has been done to create processes, procedures, or resources generated by the implementation plans. The same survey will be sent to clinicians three times: before the start of active implementation, 3 months into the implementation process, and at the end of the implementation process (6 months). For the CBT+ Dashboard, we will work with the CBT+ program to extract data on nuanced elements of MBC fidelity consistent with the IMPACT Center’s overarching Quality Model [38, 39], and our emphasis on using MBC to improve treatment quality for youth with comorbid mental health problems. See Fig. 7 for the IQM, which was adapted from the seminal paper from Proctor and colleagues [38].

#### Participants

We will ask all clinicians that work with youth and have consented to study activities to complete the clinician self-report. For fidelity monitoring using the CBT+ Dashboard, we will recruit up to five clinicians from each clinic. Each clinician will enter data for 3–5 youth clients into the CBT+ Dashboard. All eligible clinicians from the clinics and their clients from 2 years prior to this study will be historical controls (~3 clinicians per year with data for 2–4 clients, for a total of ~108 control clients).

#### Measures

The clinician self-report measure is a simple, quick, and homegrown self-report tool based upon the Collect, Share, Act conceptualization of MBC fidelity [8]. The creation of this survey was guided by a need for something brief and pragmatic, and no self-report measures to our knowledge are (1) brief, (2) pragmatic, and (3) assess fidelity to the three main components of MBC [40]. The survey asks clinicians to report (1) their approximate youth caseload in the last 6 months, and the percentage of youth patients with whom they have (2) collected data at least every two sessions, (3) shared data over the treatment course, and (4) acted on these processes to change the direction of treatment. Clinicians will complete this survey over REDCap [37]. We plan to assess the internal reliability, concurrent validity, and convergent validity of the measure once data collection ends [41]. A copy of this measure is available from the first author.

For fidelity tracking using the CBT+ Dashboard, clinicians will enter relevant youth and/or caregiver self-report measures into the CBT+ Dashboard when administered. Measures included in the CBT+ Dashboard include the following: a trauma exposure screen and posttraumatic checklist, a general assessment of symptomology (internalizing, externalizing, and attention symptoms), depression, and anxiety, all expected to be administered at baseline and personalized through the course of treatment [16].

All clinic partners involved in rapid ethnography will also complete the Implementation Climate Scale at baseline (ICS; [42]). The ICS is an 18-item survey that assesses the expectations, supports, and rewards associated with evidence-based practice implementation in an organization. The ICS has been used extensively in implementation studies in behavioral/mental health settings and has demonstrated solid internal consistency and structural, convergent, and discriminant validity [42–45].

#### Analytic plan

We will use 3-level generalized mixed effects models (fidelity score nested within clinician nested within clinic) to assess whether tailored implementation plans led to significant changes in MBC fidelity. The ICS will be treated as a covariate. Only effects at the clinic level are considered fixed, as this is a nuisance cluster. Each predictor will be modeled separately, with appropriate link functions for distributional form and dichotomous or count variables. Significance of model fit and individual coefficients will be determined via deviation tests (likelihood ratio, Akaike Information Criterion, and Bayes Information Criterion).

### Aim 3: Co-design toolkits for IMPACT methods piloted in Aim 1 via UCD workshops

Following the implementation process, we will co-design toolkits for IMPACT methods with practice partners, primarily for methods that clinic staff could actually use: barrier prioritization and design kits. Toolkits will consist of a brief history and rationale for a specific method and provide practical considerations, actionable steps, and resources for using that method to achieve a specific goal. Toolkits are intended to be publicly available, practical, and usable. Co-design with practice partners will consist of a focus group followed by a user-centered design group to gather feedback on toolkit structure and content for all available toolkits.

#### Participants

For UCD with practice partners, we will recruit *n* = 12 participants to participate in two design groups, one for barrier prioritization and one for design kits (total *N* = 24). To be eligible, participants could have been involved in any study activity.

#### Procedure

First, methods core team members will work with communications and visual experts to develop each toolkit based on the workshop design input and present it to practice partners via email to solicit additional asynchronous feedback for iteration before publishing the toolkits online. Once the toolkits have been created, an IMPACT Center UCD expert will lead the 2-h virtual workshops with practice partners. In the first 40 min, workshop participants will be prompted to remember their experience working with the NIMBLE team. For the next 30 min, workshop participants will receive the method toolkit, an overview of workshop objectives, and prompts to inform their engagement with the materials. The next 40 min will consist of facilitated discussion of the reviewed toolkit. Participants will consider toolkit materials using prompts like: “How effectively do you think you could follow [example toolkit] in its current form?” A co-facilitator will capture feedback that informs revisions/refinements to methods or toolkit design. In the final 10 min, we will debrief and engage in member-checking to ensure the accuracy of the feedback we obtained. If conflicting feedback arises, the facilitator will work to understand the conflict source and help participants reach a consensus by clarifying common goals for the toolkit.

#### Analysis

Focus group feedback will be formally analyzed and organized by a priori established themes (e.g., acceptability, feasibility) and emergent themes, both populated with direct quotes to ensure the accuracy of data capture and sharing. We will use thematic analysis on transcript data to identify actionable improvements to the barrier prioritization and design kit toolkits. We will administer the Acceptability of Intervention Measure, Intervention Appropriateness Measure, and Feasibility of Intervention Measure to supplement the interpretation of qualitative findings [46, 47]. 

## Discussion

MBC holds promise for improving the impact of psychotherapy for youth, especially in community mental health centers where comorbidities and clinical complexity are common. To overcome suboptimal implementation of MBC, tailored implementation approaches that tackle the critical barriers are needed. The NIMBLE study pilots multiple practice partner-engaged methods that center the voices of those “on the ground” to create nuanced, locally contextualized implementation plans to improve MBC fidelity. To identify barriers, we will use rapid evidence synthesis, design kits, and rapid ethnographic assessment. To prioritize barriers, we propose a novel, group-facilitated barrier prioritization exercise. To co-create implementation plans that are guided by the voices of practice partners, we will use a group-facilitated causal pathway diagramming method. We will evaluate whether fidelity was optimized in these settings as a result of these exercises and engage practice partners to understand their perceptions of our methods and to further improve our methods.

### Anticipated challenges and limitations

We anticipate several challenges. Community mental health centers are under enormous pressure and have few resources. The landscape of doing large-scale implementation studies in community mental health centers has clearly been affected by the sequelae of the COVID-19 pandemic. Turnover is common [48] and clinicians are under unprecedented amounts of stress [49]. We expect to have difficulty recruiting clinics and clinicians as well as clinician attrition. Indeed, several clinics that offered letters of support were no longer able to participate when we approached them.

We also anticipate challenges while piloting novel, untested methods. For instance, we anticipate difficulty with getting participants to return design kit materials. Additionally, design kits have inherent accessibility limitations; youth who are visually impaired or have disabilities that limit writing capabilities are excluded automatically from this method. So while design kits promise to increase equitable implementation practices, researchers should make efforts to incorporate design kit methods that allow for text-to-speech. Over the course of this study, we will make efforts to include other response options for participants, including voice memos and digital text. Finally, given that design kits have never been used in implementation research, it is possible that the data gleaned from them is not relatively beneficial in comparison to other methods like REA.

This study has several important limitations. First, we are limited in the types of implementation support we can provide to practice partners. For instance, if practice partners use a specific electronic medical system to do MBC, and many of the identified MBC barriers are related to the interface of that system, we may not be able to make significant changes to those established systems. Second, the very nature of rapid methods means that some steps of scientific rigor are eliminated. For instance, while every effort will be made to gather every relevant study in the RES, it is possible that we will miss some studies.

## Conclusion

Implementation science and practice have historically minimized the voices of those outside of positions of power [50]. Practical and freely accessible methods that meaningfully engage practice partners could have a transformative impact on mental healthcare in these clinics and beyond by making it easier to engage in rigorous and effective implementation of MBC and other EBPs.

### Supplementary Information

**Additional file 1.** Design kit prompts.

## Data Availability

Not applicable.

## Abbreviations

AIM: Acceptability of Intervention Measure
CBT+: The Washington State CBT+ initiative
CMHC: Community mental health center
COVID-19: Coronavirus disease of 2019
CPD: Causal pathway diagram
EAGLES: Enhanced Art, Graphics, Literacy, & Editorial Strategies
EBP: Evidence-based practice
FIM: Feasibility of Intervention Measure
IAM: Intervention Appropriateness Measure
ICS: Implementation Climate Scale
MBC: Measurement-based care
MeSH: Medical Subject Headings
NIMBLE: Novel Methods for Implementing Measurement-Based Care in Low-Resource Environments
PHQ-9: Patient Health Questionnaire-9
REA: Rapid ethnographic assessment
RES: Rapid evidence synthesis
UCD: User-centered design
