# Supplementary material for: Study protocol: Novel Methods for Implementing Measurement-Based Care with youth in Low-Resource Environments (NIMBLE)
Source: Implement Sci Commun. 2023 Nov 28;4:152. doi: 10.1186/s43058-023-00526-z (PMC10683142; doi:10.1186/s43058-023-00526-z)
Supplement: Supplementary file 1 — Additional file 1. Design kit prompts. [file 43058_2023_526_MOESM1_ESM.pdf]

INSTRUCTION CARD

Start Here

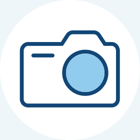

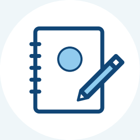

Please find the 7 activity cards.

They are numbered in the order that you should do them, for example activity 1 on the first day, activity 2 on the second day.

The card will tell you to use the camera, the journal, or both. Some activities may be more important to you than others, so you might spend more time on them. **There are no right or wrong answers.**

Questions?

Call the Project Manager, Carolyn Bain, at 206-287-4611 or Dr. Ruben Martinez at 206-287-4618.

1

Activity Card

Show and tell us more about where you work.

Include photos of places where you spend time with your coworkers, rooms where you do therapy, shared workspaces, the front desk, and places where you go to find peace and quiet.

Tell us about the pictures you took. Include details to help us understand where you work.

1

Please remember not to include any names, places, or other information that would identify you or your patients.

2

Activity Card

Show us your personal workspace or office. Please include photos of your desk in the morning, afternoon, and evening.

Tell us how you feel about your office, including issues that come up with the way your workspace is organized.

Do you enjoy being in your office? Does your space feel adequate? Do you do therapy in your office?

2

Please remember not to include any names, places, or other information that would identify you or your patients.

3

Activity Card

We want to understand your workflow using measurement-based care (MBC). Take photos of the steps you take to do MBC, and the places where you do MBC.

Examples are a photo of the software you use to enter de-identified scores, places where you find measures (e.g., cabinets, closets), places where measures are administered and scored, and other steps of the measurement process.

Describe your photos and tell us how and when MBC is part of your workday.

How often do you think about MBC? Is MBC part of your day-to-day and week-to-week activities, or are there specific days and times when you think about MBC?

3

Please remember not to include any names, places, or other information that would identify you or your patients.

4

Activity Card

Tell us how you would make measurement-based care (MBC) better. What changes to your day-to-day and week-to-week workflows would you make, and how would you change them?

If you aren't sure how to answer, these questions might help you get started. You do not need to answer every question:

1. Does the way you are supposed to do MBC make sense to you?
2. Are measures accessible to you? If not, where would they be located?
3. Are clinical cutoffs for measures available to you? If not, where would they be?
4. Is there anything about the measures themselves that you do not like? Are they appropriate for your patients? Are they too long? Are they age-appropriate?

4

Please remember not to include any names, places, or other information that would identify you or your patients.

5

Activity Card

Tell us about a time when you or your clients felt excited or positive about using or discussing measurement-based care, preferably in the last week.

Examples could include a patient whose scores on measures improved significantly, a significant change from measurement-based care that improved treatment, or something else. If that has not happened in the past week, share an example from another time.

5

Please remember not to include any names, places, or other information that would identify you or your patients.

6

Activity Card

Tell us about a time when you or your clients felt discouraged or negative about using or discussing measurement-based care, preferably in the last week.

Examples could include a patient whose scores didn’t change, or a conversation about progress measures that didn’t go as planned. If that has not happened in the past week, share an example from another time.

6

Please remember not to include any names, places, or other information that would identify you or your patients.

7

Activity Card

Tell us about the emotions and thoughts that come up for you when you are doing measurement-based care during a client crisis, including before you give a measure, while you score measures, while you enter them, and/or when discussing results with colleagues, supervisors or clients.

7

Please remember not to include any names, places, or other information that would identify you or your patients.

INSTRUCTION CARD

Start Here

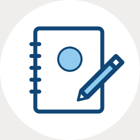

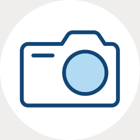

Find the 7 Activity Cards. Each card has an activity.

Cards are numbered in the order that you should do them. Please do activity 1 on the first day, activity 2 on the second day, and so on.

The card will tell you if you need to use the camera, the journal, or both. Include as much detail as you want, and share anything that helps you express yourself, such as poems and drawings.

You might have more to say about some activities than others. If some questions are hard to answer, we included more questions to help you get started. You can skip part of an activity if you don't want to do it for any reason. **There are no right or wrong answers.**

Questions?

Call the Project Manager, Carolyn Bain, at 206-287-4611 or Dr. Ruben Martinez at 206-287-4618.

1

Activity Card

Tell us about your daily routine.

Take photos that help us understand your daily routine. You might take photos of things you do each day, such as chores, hobbies, and other routines.

You might take photos of places you do those things.

We want to understand what it is like to live your life. Use the journal to tell us why you took each photo.

1

Remember! Do not write about or take photos of anything that could identify you, your family, or anyone else.

2

Activity Card

Tell us about the places where you spend the most time. What about those places is special or meaningful?

Take photos of these places.

2

Remember! Do not write about or take photos of anything that could identify you, your family, or anyone else.

3

Activity Card

How do you usually feel before, during, and after you go to therapy? What do you think about before, during, and after you go to therapy?

Take photos of how you feel before, during, and after therapy.

For example, your energy level might change after therapy. You could take photos of what you did after your therapist visit.

3

Remember! Do not write about or take photos of anything that could identify you, your family, or anyone else.

4

Activity Card

What kinds of things make engaging in therapy hard for you?

Examples might be: homework, activities outside of school such as sports, or something about therapy or the clinic you go to.

Take photos that show how or why these things make therapy hard.

4

Remember! Do not write about or take photos of anything that could identify you, your family, or anyone else.

5

Activity Card

What is it like for you to fill out questionnaires about how you have been feeling lately?

Flip this card over to see an example of a questionnaire.

If you do not know how to answer this question, these questions might help you get started. You do not need to answer every question:

1. Did answering these questionnaires feel helpful or unhelpful?
2. What thoughts or feelings do you have when you fill out these questionnaires?
3. Are these questionnaires important to you?
4. Has your therapist ever talked about why you do the questionnaires?
5. What would you change about these questionnaires or questions?

5

		0<br>Not True or Hardly Ever True	1<br>Somewhat True or Sometimes True	2<br>Very True or Often True
1.	When I feel frightened, it is hard for me to breathe	○	○	○
2.	I get headaches when I am at school	○	○	○
3.	I don’t like to be with people I don’t know well	○	○	○
4.	I get scared if I sleep away from home	○	○	○

Remember! Do not write about or take photos of anything that could identify you, your family, or anyone else.

6

Activity Card

Tell us about a time when you felt much better or much worse because of therapy.

If you do not know how to answer this question, these questions might help you get started. You do not need to answer every question:

1. Do the questionnaires reflect how much better or worse you feel?
2. Would you talk to your therapist about whether you are feeling much better or worse? Why or why not?
3. What questions could your therapist ask you to find out if your problems were getting much better or worse?
4. How would you or your therapist know that you need to make a change in therapy?

6

Remember! Do not write about or take photos of anything that could identify you, your family, or anyone else.

7

Activity Card

Tell us about the kinds of activities or topics that have helped you in therapy.

If you aren't sure how to answer this question, these questions might help you get started. You do not need to answer every question:

1. What kind of activities in therapy have helped you improve your life?
2. What kinds of activities (games, conversations, questions) or topics (thinking about thoughts, learning to relax, doing "practice") work better for you than others?
3. When things you do in therapy feel helpful or unhelpful, do you tell your therapist? Why or why not?

7

Remember! Do not write about or take photos of anything that could identify you, your family, or anyone else.
